# Examining concurrent validity and item selection of the Session Wants and Needs Outcome Measure (SWAN-OM) in a children and young people web-based therapy service

**DOI:** 10.3389/fpsyt.2023.1067378

**Published:** 2023-02-09

**Authors:** Santiago De Ossorno Garcia, Julian Edbrooke-Childs, Louisa Salhi, Florence J. M. Ruby, Aaron Sefi, Jenna Jacob

**Affiliations:** ^1^Kooth Plc, London, United Kingdom; ^2^Anna Freud Centre, CORC, London, United Kingdom; ^3^Evidence Base Practice Unit (EBPU), University College London, London, United Kingdom; ^4^School of Psychology, University of Kent, Canterbury, Kent, United Kingdom; ^5^Department of Psychology, University of Exeter, Exeter, Devon, United Kingdom

**Keywords:** single session therapy (SST), instrument evaluation, digital mental health, web-based therapy, internet delivered psychological treatments, concurrent validity, patient reported outcome measures (PROM), SWAN-OM

## Abstract

**Background:**

Single-session mental health interventions are frequently attended by children and young people (CYP) in both web-based and face-to-face therapy settings. The Session “Wants” and “Needs” Outcome Measure (SWAN-OM) is an instrument developed in a web-based therapy service to overcome the challenges of collecting outcomes and experiences of single-session therapies (SSTs). It provides pre-defined goals for the session, selected by the young person prior to the intervention, on which progress toward achievement is scored at the end of the session.

**Objective:**

The objective of this study was to evaluate the instrument's psychometric properties, including concurrent validity against three other frequently used outcome and experience measures, at a web-based and text-based mental health service.

**Methods:**

The SWAN-OM was administered for a period of 6 months to 1,401 CYP (aged 10–32 years; 79.3% white; 77.59% female) accessing SST on a web-based service. Item correlations with comparator measures and hierarchical logistic regressions to predict item selection were calculated for concurrent validity and psychometric exploration.

**Results:**

The most frequently selected items were “*Feel better*” (*N* = 431; 11.61%) and “*Find ways I can help myself*” (*N* = 411; 11.07%); unpopular items were “*Feel safe in my relationships*” (*N* = 53; 1.43%) and “*Learn the steps to achieve something I want*” (*N* = 58; 1.56%). The SWAN-OM was significantly correlated with the Experience of Service Questionnaire, particularly the item “*Feel better*” [rs_(109)_ = 0.48, *p* < 0.001], the Youth Counseling Impact Scale, particularly the item “*Learn the steps to achieve something I want*” [rs_(22)_ = 0.76, *p* < 0.001], and the Positive and Negative Affect Schedule, particularly the items “*Learn how to feel better*” [rs_(22)_ = 0.72, *p* < 0.001] and “*Explore how I feel*” [rs_(70)_ = −0.44, *p* < 0.001].

**Conclusion:**

The SWAN-OM demonstrates good concurrent validity with common measures of outcome and experience. Analysis suggests that lesser-endorsed items may be removed in future iterations of the measure to improve functionality. Future research is required to explore SWAN-OM's potential to measure meaningful change in a range of therapeutic settings.

## 1. Introduction

In the field of digital mental health products and services, where the aim is often to increase access to services and provide choice and flexibility, brief and sometimes only one session is often the common engagement form of professional support. Digital mental health services are well placed to deliver brief and focused interventions with human-mediated support as well as evidence-based and self-guided programs (1, 2). Web-based scalable services and interventions are important tools to tackle demand by increasing access to preventative and early intervention support for children and young people's (CYP) mental health.

Children and young people as users engaging with web-based services will often mimic face-to-face services with a single-session engagement, with one-off sessions being the most frequent across services (3, 4). The opportunities to examine the changes and monitoring of interventions in the digital healthcare context are promising, due to tracking information technology, ease and quick access to the intervention, and large data volumes that can be collected and analyzed quickly (5). A digitally enabled intervention of web-based, single-session therapy (SST) is a good starting point to understand how change takes place in this intervention and to continue to collect evidence about the effectiveness and impact of SST.

### 1.1. What are single-session therapies

Single-session therapies or one-at-a-time (OAAT) approaches (6) are interventions delivered by practitioners across a range of settings and psychological support services. SST and OAAT approaches are broad, and they have been defined as “*A purposeful endeavour where both parties set out with the intention of helping the client in one session, knowing that more help is available if needed*” (7). SST is conducted by professionals who seek to use their existing therapeutic skillset to address the presenting concerns or problems within one session and assumes that support will not be ongoing over several weeks or months (8). SST uses a strength-based approach to help clients; the focus lies on client-led “in-session” goals rather than longer-term therapeutic goals, making the most of someone's circumstances. This type of support lends itself to a person-centered approach in which objectives and outcomes are client-led, rather than a manualized course of therapy outcomes (9). In most services offering SST, like in traditional walk-in therapy (10), people access the service at the point of need, and no appointment is necessary to receive support. Additional support is offered alongside SSTs and other brief interventions (11, 12), or services may offer further support that the individual can use later (e.g., signposting advice, further counseling sessions, group sessions, and referrals). Some use the term “*one-at-a-time*” (13) to avoid misunderstanding or resistance by the clinical community that SST means only once; in a single session and other brief interventions, the study can continue beyond the implicit one-time misconception often attributed to the SST term, so both terms SST and OAAT, despite some differences, often imply that more support is available and people can access it “*at the time of need*” (6).

Single-session therapies and brief interventions are gaining momentum and adoption among mental health services, especially for young populations. Brief interventions like SST may help address the unmet needs of people waiting for services and meet the steady increase in demand for mental health support in recent decades. This increased demand calls for a transformation of services through novel models of effective delivery, including SST and brief approaches (14, 15). For example, meta-analytic evidence suggests that single-session-targeted interventions can be effective in anxiety reduction, behavioral problems, and substance use (16–18). However, single-session interventions, or SST, have not always been considered a meaningful and effective type of therapeutic intervention. This is partially due to the assumption that one session may indicate dissatisfaction or service drop-out, as the client has not finished their course of therapy or assessment as far as the design of the practice is concerned. This is, however, more difficult to discern for internet-delivered and web-based interventions (19), especially if the SST model is not explicit. It has taken some time for the field of counseling to recognize SST as a relevant therapeutic intervention (20). Evidence from walk-in therapy clinic studies worldwide (21) has revealed the acceptance of SSTs, and evidence from some studies reported that 70–95% of people receiving SST were satisfied with their sessions (3, 22, 23). A further trial reported that one session was perceived as enough at the clinics when offered with support (24). Single sessions and brief interventions are important to be examined, as they may be not only a cost-effective way to increase access and provide a scalable solution for the rising demand for mental health support but also a way to understand the needs and access of the population that mental health services are intending to serve.

Single-session therapy has been previously recommended as a treatment of choice for CYP presenting with mental health difficulties (23) and has the potential to be one of the drivers for system change and transformation described in the “THRIVE” framework to support CYP's mental health (25). Moreover, the National Health Service (NHS) in the UK has made a move to accept one-session interventions as one of the potential changes that may help to tilt the needle on waiting times for improving access to psychological therapies (26); this supports the wider popularity of SST and its recognition as a therapeutic intervention. This is further supported by evidence from Children and Adolescent Mental Health Services (CAMHS) in the UK, where one session is the most common way to engage with services (27). In addition, SST may be preferred by certain groups of young people when accessing therapy, namely, those who value choice and flexibility when receiving support; these are two clearly defined factors for pluralistic and accessible psychotherapy provision (18, 23, 28). One-session engagements from specialist mental health services at CAMHS are evidenced as being most commonly attended by young people with complex needs, from minoritized ethnic groups, with relational difficulties with peers, and also by those with less frequently occurring problems; this evaluation, however, did not examine SST in their evaluation of engagement (27). Therefore, it is yet to be known how useful it will be to implement tailored therapies like SST as the most common form of engagement in services and its effectiveness and outcomes to individuals.

### 1.2. Outcome measurement for single-session therapy

A range of outcome measures is used to measure SST effectiveness (29–31). These often are targeted to specific mental health difficulties (e.g., anxiety and depression) such as the Revised Children Anxiety and Depression Scale (1), Pediatric Anxiety Rating Scale (32), or Counseling Progress and Depth Rating Instrument (33, 34), among others. However, there is a prevailing challenge related to the short-term nature of SST and the disconnect with the longer-term measurement of mental health difficulties. Authors from one study have also emphasized the importance of measuring immediate changes after SST (18). On the one hand, SST seems to be influenced by unspecific factors to the overall success of SST and its impact on change score and clinical improvement (35). On the other hand, SST is not a treatment modality that easily enables the measurement of change over several time points (36). Although there is a growing effort and evidence to demonstrate clinically significant improvement over time with controlled studies (17, 37, 38), most of them showed short-term improvement only after 1 month and failed to demonstrate improvement in further follow-ups (39, 40). Other outcome instruments for psychotherapy are designed for monitoring, where a course of therapy is assumed, which does not match with the immediacy of the SST framework, where using a series of scores to monitor change is not expected or instruments that ask temporal questions about problems (e.g., “*…in the last two weeks*”) such as the YP-CORE and CORE (41) may be inadequate to assess the here and now. This also raises challenges when embarking on testing the reliability (in terms of measurement error) of SST outcome measures. SSTs often assume that ongoing sessions are not required for improvement (38). This emphasizes why it may not be possible, or appropriate, to examine the test-retest and repeated measurements in SST outcomes, as these should be related directly to the session outcomes and experience of the intervention, rather than something re-measurable at a later measurement point.

In pluralistic services, in particular, the therapeutic background and practitioner perceptions can influence the course of therapy, allowing different therapeutic approaches to be used in the SST; thus, it is difficult to systematize or explain the components that lead to its effectiveness (42). Overall, tracking progress from SSTs can be difficult, and further follow-up with the young person is not obtained nor sought by providers regularly; thus, missing data will appear frequently when obtaining longitudinal data outside of controlled studies. There is also a further challenge of capturing personalized outcomes and goals, which complement the pluralistic nature of SST work due to the brevity of these interactions. Balancing the need for a short, tailored measure, this can serve not only as an outcome within the brief nature of a single session but also as a measurement instrument that helps to focus the brief encounter and maximize time working with the person-chosen goals.

The suggested solution, which addresses the challenges highlighted, is a patient-reported outcome measure that captures the individual “Wants” and “Needs” of the single session. These “Wants” are intended to represent the choice of common goals expressed and to be achieved within one session. The instrument also covers the “Needs” as psychological nutrients that are required to grow, foster wellbeing, and achieve meaning in life (43). There is a tension between what CYP, as clients desire and “Want” and what they “Need” from therapy (44), and indeed studies suggest that people are not always certain of what is best for them while in therapy (45). However, directive “Needs” may assume an expert approach to CYP, bringing institutional or authoritative representation into delivering support and increasing the chances of mistrust and disengagement, which may contradict the person-centered delivery. Therefore, it is important that people and therapists can identify the basic psychological needs regarding how to foster autonomy, competence, and relatedness (46, 47) within one session and quickly enable a solution-focused support so the right help can be provided at the point of need. It is a balancing act in the support intervention by exploring “Wants” and “Need”, so people understand and feel they are participating in the choice for support while understanding that more help is available if needed, as well as setting a framework to provide a positive and safe space to influence growth mindsets in CYP (1).

This highlights the importance of delivering a pluralistic and person-centered intervention in SST and highlights that the design should hold those principles in mind to achieve balance in a directed and person-centered intervention like SST. The instrument also assumes that to be able to obtain SST outcomes (a meaningful measurement of change), the session goal expectations should also be led and personalized by the client (48). Therefore, alignment between the practitioner and patient-therapeutic outcome expectations is critical when providing SST, especially when the session aims and focus have been identified by both parties. Practitioners often need to assess if indeed these expectations brought to SST are realistic for this type of presenting concerns or problems alongside monitoring any disclosure or indication for risk of harm and safeguarding. An instrument that sets a limited number of “in-session” goals can help develop this alignment as well as enhance the delivery of SST in web-based services. The SST measure provides a solution to collect, in a systematic way, aggregated SST outcomes for services delivering SST (49). As a patient-reported instrument, it also provides the client with choice by giving control over what they expect to cover in SST and introducing the ability to personalize these “Wants” and “Needs” if preferred by the user.

### 1.3. SWAN-OM as an instrument for single-session measurement

To address the need for a tailored measure to track single-session therapeutic outcomes, a new instrument contextualized for SST was required; the “*Session Wants And Needs Outcome Measure*” (SWAN-OM) was developed in a digital web-based mental health service delivering SST and OAAT approaches *via* text-based synchronous messages. The measure was originally developed in a four-phase design to examine the content and face validity of the measure aimed at CYP (aged 10–25 years), including a pilot of the measure and usability testing with relevant stakeholders, including practitioners and diverse groups of young people (50).

The instrument provides service users with a list of “Wants” and “Needs” to choose from, alongside a personalized option (“*free-text*”). The SWAN-OM has a novel format with a two-step filtering logic, where young people can select from six higher-level themes and, within these themes, specific items. Once the number of selected themes is explored, young people can select up to three items in total to cover in their SST goals. Once these “in-session” goals are selected, the practitioner can look at what the person has chosen to focus on in their SST. This gives information to the practitioner on how their intervention can be tailored to everyone, especially in the context of a digital mental health service, i.e., anonymous, free, and accessible, where users may present with a wide range of concerns and difficulties.

Outcomes and experiences after the session are measured on a Likert scale, indicating how much they achieved what they initially wanted. This instrument can determine if “Wants” and “Needs” were met throughout the SST encounter. At the end of their session, they are only asked about how much they achieved in the items they selected, rather than how much they achieved across all instrument items. This provides a novel way to measure what young people “Want” or “Need” from an SST in a web-based therapy service. It also facilitates the formulation of “in-session” goals as items for the practitioner to structure their session. The two-stage logic measure structure, going from a group of themes for selection to item display and selection of “Needs”, provides a manageable “in-session” goal-setting activity for young people; this logic structure was suggested by young people in a stakeholder workshop during its development to present the information. The SWAN-OM structure means that traditional psychometric testing may not be appropriate; however, there is an opportunity to examine this measure at an item level, which we go on to explore in this research.

### 1.4. The present study

This evaluation aims to explore the concurrent validity of the SWAN-OM using other standardized instruments as comparator measures, chosen due to relevance, similarity of items, and immediacy. The audit of the instrument further explores construct validity by predicting item selection based on population characteristics. We also discuss limitations and considerations when examining the psychometric properties of instruments that have novel structural designs, such as the SWAN-OM.

This evaluation examines the data collected in a web-based counseling service, where the SWAN-OM was administered alongside the other measures. We hypothesized that a therapeutic encounter like SST should have a positive association with positive emotional changes. Therefore, positive SWAN-OM scores would correlate positively with a positive affect scale; we also expect to see positive changes in the affect scale before and after the SST. We expect most items from the SWAN-OM to correlate positively with a session progress rating scale. Finally, we expect positive SWAN-OM scores to correlate positively with an experience or satisfaction of service measure.

## 2. Methods

### 2.1. Participants

Young people who participated in this study were users of a digital mental health service (Kooth.com). To be eligible to take part, young people had to have no previous engagement with counseling sessions within the service. Young people in the UK, commonly aged between 10 and 25 years, can access the digital mental health service online and anonymously register to receive web-based therapy services without cost. All participants for the study were required to have requested access to a synchronous text-based chat session with a practitioner in the digital service online and wait briefly until being seen by a practitioner. Data from young people were collected on the service between January 2021 and June 2021. Only data from young people using Kooth during that period who had explicitly provided research consent when using the service were available for this evaluation; no parental consent was sought for parents to preserve the safety of those aged under 16 years and anonymity during the evaluation; all users provided consent for research and evaluations at the point of registration at Kooth. Gender, age, and ethnicity were self-reported variables collected directly from the young people as part of the service sign-up process for the digital service. A service evaluation with the new measure alongside other comparator measures was deemed the best implementation approach to reduce disruption in the service (e.g., detracting people from accessing support due to research) and maintain clinical governance, as voluntary participation and information were provided prior to administering the instruments during the implementation of this instrument and the evaluation period.

Over this evaluation period, 1,401 young people accessed 1,901 chats within the service. On average, a young person accessed the chat 3.2 times during the study period (with a minimum of one and a maximum of 26 chats), and each chat lasted on average 52 min (*SD* = 21.6; extreme outliers removed). Young people who took part in the study were aged between 10 and 32 years, with an average age of 15.9 years (*SD* = 2.9). Most young people accessing the service were female (*N* = 1,087; 77.59%) from a white ethnic background (*N* = 1,111; 79.3%). In total, 1,435 (75.13%) chats included information about the participants' presenting concerns, as reported by practitioners. The majority indicated experiencing difficulties with anxiety/stress, suicidal thoughts, self-harm, and family relationships (Table 1).

**Table 1:** Demographic characteristics^a^.

**Demographic variables**	***n***	**%**
**Gender**		
Agender	47	3.35%
Female	1,087	77.59%
Gender fluid	57	4.07%
Male	210	14.99%
**Age**		
10–14	492	35.12%
15–19	788	56.25%
20 and above	121	8.64%
**Ethnicity**		
Any other ethnic group	10	0.71%
Asian/Asian British	106	7.57%
Black African/Caribbean/Black British	49	3.5%
Mixed multiple ethnic group	78	5.57%
White	1,111	79.3%
Not Specified	47	3.35%
**Presenting concerns**		
Mental Health	1,105	57.85%
External issues	734	38.43%
Suicidal thoughts/Self-harm	601	31.47%
Risk	163	8.53%
Physical/Other	83	4.35%
No information provided	475	24.87%

a Data collected from 1,401 young people attending 1,910 chats. Percentages are based on the total number of young people for the categories Gender, Age, and Ethnicity; percentages are based on the total number of chats for the categories related to presenting concerns. Percentages reported for presenting concerns do not add up to 100% as young people can be assigned more than one presenting concern per chat.

### 2.2. Instruments and variables

#### 2.2.1. Young people's characteristics

The service collects four different categories for gender (male, female, agender, and gender fluid). Age was collected as a continuous variable and divided into three age groups for analysis purposes (10–14, 15–19, and 20 years and above). Ethnicity was also grouped into five categories as recommended by the ONS with the available service data (51).

#### 2.2.2. Session wants and needs outcome measure

The SWAN-OM is a 21-item single-session outcome measure (see Supplementary material Appendix section); the face and construct validity of the instrument have been previously examined as part of its development and design within the digital service (50). Young people are presented with the SWAN-OM prior to the intervention (pre-chat item selection) and immediately after the intervention (post-chat item scoring).

**Figure 1:**
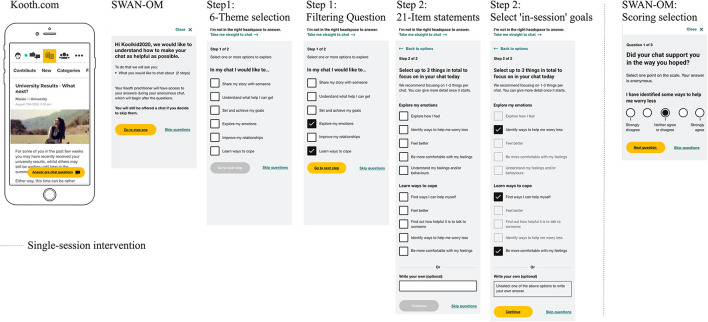
Smartphone wireframes of SWAN-OM at Kooth.com.

The instrument follows a two-step logic:

- First, the young person is asked to select any of the following: “In my chat I would like to...” (A: “*Understand what help I can get*”; B: “*Share my story with someone*”; C: “*Set and achieve my goals*”; D: “*Explore my emotions*”; E: “*Improve my relationships*”; F: “*Learn ways to cope*”).
- Second, depending on the theme selection, the 21 items from SWAN-OM are displayed after a “*Select up to 3 things in to focus on in your chat today*” prompt that reflect their aims for the chat session. These are seen as “in-session” pre-defined goals tailored to the “Wants” and “Needs” reported by the young person at the point of access to the session (Figure 1).
- After the SST intervention (post-chat item scoring), young people are again presented with the instrument and asked, “*Did your chat support you in the way you hoped?*” to indicate how much progress they had made on each, using a 5-point Likert scale ([-2]: “*strongly disagree*” to (2): “*strongly agree*”) with follow-up statements that match what was selected prior to the session (refer to statements in the Appendix).
- One of the 21 items is a free-text option (write your own) for personalization. For this personalized item, young people are presented with the following text at the post-chat item-scoring stage: “*I chose to write my own focus before the chat and I was supported the way I hoped*.”

#### 2.2.3. Positive and negative affect schedule

The Positive and Negative Affect Schedule (PANAS) (52) was selected as an instrument due to its wide use in the research literature and the immediacy of measuring emotional states as a proxy for the quality of the SST therapeutic encounter measured before and after the session. The PANAS is a measure of affect that can be used with children aged 6–18 years (53). It has been extensively validated in different languages, showing excellent psychometric properties, and it is the most widely used instrument to measure affect (54). It includes 10 items assessing affect in the present moment and is divided across two subscales: Positive Affect (PANAS-PA) and Negative Affect (PANAS-NA). The following 10 feelings are used: Sad, Happy, Scared, Miserable, Cheerful, Proud, Afraid, Joyful, Mad (Angry), and Lively. PANAS assesses a person's positive and negative trait affect using a 5-point scale (1 = “*very slightly or not at all*”; 5 = “*extremely*”). The schedule has been validated in general and clinical populations (52), which makes it a suitable instrument to use in a digital service where clinical and non-clinical populations are accessing the service. In the current evaluation, PANAS was administered at Time 1 (pre-chat) and Time 2 (post-chat) before and after the single-session intervention. In our sample, both Negative and Positive subscales showed good internal consistency at Time 1 and Time 2: Negative subscale, α = 0.75 (Time 1) and α = 0.84 (Time 2); Positive subscale, α = 0.82 (Time 1) and α = 0.90 (Time 2).

#### 2.2.4. Youth counseling impact scale

The insights subscale of the Youth Counseling Impact Scale (YCIS) (55, 56) aligns to measure the impact of the session, or perceptions of having made progress within a session, which are also associated with clinical treatment outcomes (57–59). The YCIS is a 6-item scale assessing young people's perceptions of the impact of individual mental health counseling sessions on their thoughts, feelings, and behaviors. The YCIS was designed to be used with people aged 11–18 years and showed good psychometric properties (60). The original instrument is divided into two subscales: insights, assessing impact immediately after the session, and change, measuring the impact of the 2 weeks following the session (61). The 3-item insight subscale was selected for the service improvement evaluation; in this subscale, young people indicate how well each item reflected the outcome of the session using a 5-point Likert scale (1 = “*Not at all*”; 5 = “*Totally*”). The insight subscale showed good levels of internal consistency based on Cronbach's alpha α = 0.86 during the evaluation.

#### 2.2.5. Experience of service questionnaire

The Experience of Service Questionnaire (ESQ, formerly CHI-ESQ) (62) was selected for comparison with SWAN-OM after the SST took place in order to understand the satisfaction that the user had with the service and with the care provided. The instrument is used across mental health CAMHS services for quality and experience assessments and evaluations. The ESQ is a 12-item questionnaire that can be used with young people aged 12–18 years to measure feedback about a mental health service and, concretely, satisfaction with care and the environment; it is commonly used for CAMHS services to measure the subjective experience of satisfaction (63). The 9-item “Satisfaction with Care” subscale was used. Some items were adapted and rephrased to match the context; for example, “*Overall, the help I have received on Kooth (the service) is good*.” Young people rated each item on a 4-point Likert scale (1 = “*Certainly true*”; 3 = “*Not true*”). Items scored as 4 = “*Don't know*” were treated as missing data. Responses were reversed before the total score was calculated so that a higher ESQ score indicated higher service satisfaction. The internal consistency of the ESQ Satisfaction with Care subscale showed good levels, with a high-reliability score based on Cronbach's alpha α = 0.87.

### 2.3. SWAN-OM evaluation procedure

The instrument was implemented and evaluated for a period of 6 months (January 2021 to June 2021) at Kooth.com, a web-based therapy service based in the UK, *via* synchronous text messaging. The service is anonymous at the point of entry and provides person-centered text-based SST or drop-in, one-at-the-time therapy that is free and accessible to CYP in most of the UK with access to an internet connection, and who wish to register and use the service.

During the evaluation period, the SWAN-OM was implemented for SSTs. A total of 120 practitioners from the web-based therapy service were recruited and trained to administer the SWAN-OM at Kooth.com. Each practitioner attended a training session of 60 min and was provided with a manual containing guidance on how to use the instrument in the platform, internal clinical governance procedures, SST relevant literature, and frequently asked questions about the instrument and the research study. *Ad hoc* support was provided through instant messaging software by the research group to each practitioner. All practitioners involved in the study were part of the service workforce. Therefore, practitioners were in training or had obtained their counseling or clinical qualifications as mental health practitioners. The service holds a pluralistic view on their training and therapeutic background but all within encompassing a person-centered framework to deliver care.

The SST intervention was delivered over a 40–60 min text-based chat in the online synchronous messaging system of the web-based service. The broad SST aims were to engage, conduct a brief assessment, and meet the needs of young people where possible. Brief risk assessments and safeguarding protocols were prioritized above those aims as part of service provision; these include a routine risk inquiry in every chat with questions to users explicitly asking about harm to themselves or others, and, if there is a disclosed risk, the single-session appeared will follow safety procedures as opposed to the SWAN-OM selected “Wants” and “Needs” and its processes. The approach of SST delivery by practitioners within the service was pluralistic (64) with a broad range of therapeutic orientations. The SST interventions delivered during the evaluation considered the brief-intervention mindset and its blend with traditional approaches to counseling (65), in addition to the already established evidence based on SST (13, 18, 66).

The SWAN-OM was administered when practitioners clicked a button in the platform to launch the questionnaires in the front-end view of the user. The battery of instruments was administered at the same points in time, before the chat (Time 1: pre-SST; PANAS and SWAN-OM) and after the chat (Time 2: post-SST; PANAS, YCIS, ESQ, and SWAN-OM).

Young people could skip the measures if they wished to at the time of accessing the service. The practitioner was able to access the item selection of the SWAN-OM at Time 1 and then start the SST when ready. Following the end of their SST chat with a practitioner, young people were asked to complete the post-session measures. Individuals who skipped the administration of the questionnaires at Time 1 were not presented with the other measures at Time 2.

### 2.4. Statistical analysis

The dataset was cleaned and analyzed using R open-source package and language for statistical computing (67). Completion rates for the SWAN-OM and the other comparator measures alongside descriptive statistics were calculated for the audit. To investigate the psychometric properties of the SWAN-OM, different analytical strategies were followed, taking into consideration the structure of the instrument, the sample size availability, and suitability of the analyses conducted during the evaluation. Pair-wise comparisons across the administered measures were performed to investigate the concurrent validity of the instrument using correlations, and regression models were computed to understand participant characteristics and item-selection responses for the SWAN-OM.

#### 2.4.1. Analysis considerations

Considerations were made regarding the analysis, given the nature and structure of SST and the instrument. The SST outcomes based on the patient's expectations will not be repeated in a second session (even if the patient did return), as the “in-session” goals for the SST can only take place in the present moment and may not be continued in the future. Therefore, while high face validity may be achieved, it may be a challenge to obtain traditional forms of internal and structural psychometric validity, as re-testing the measure will not measure the same “Wants” or “Needs” again and, therefore, would be measuring a different SST outcome.

It was important to understand how the personal characteristics of users affected their selection of “Wants” and “Needs” for the session. This is especially important for the SWAN-OM, as item selection is shown through two-stage logic filtering of items based on the young person's theme selection. The items initially selected in the SWAN-OM should help identify if the instrument works in similar ways across a diverse cohort of young people, gain information on the most frequent “Wants” and “Needs” for SST requested by CYP at the service, and whether their characteristics predict the type of items people will select for their SST.

#### 2.4.2. Predicting item selection

Two approaches were used to assess the association between young people's demographic characteristics and item selection on the SWAN-OM. The first approach used multilevel logistic regressions, predicting items selected by young people's gender, age, ethnicity, and presenting concerns. Multilevel regression accounts for the nested nature of the data, whereby young people can access several chats and may complete SWAN-OM more than once. Log-likelihood ratio tests were used to determine whether adding demographic variables available to the model explained a significantly larger amount of variance. We used the larger category as a reference (e.g., females were used as a reference when exploring the impact of gender in item selection).

The second approach used chi-square tests to examine demographic characteristics associated with item selection to complement the multilevel regressions, especially when sample sizes were too small for specific themes or items. This occurred when small cell sizes were found in the different demographic characteristic variables and in the item selected vs. the item not selected (which would be anticipated given young people were invited to select up to three items per session). A subsample of 930 young people attending 1,131 chats was used to analyze the data; cases were discarded if young people had missing data for their demographic variables.

#### 2.4.3. Concurrent validity

Concurrent validity was explored by examining correlations between scores on individual SWAN-OM items and total scores on the comparator measures (YCIS, ESQ, and PANAS), as well as individual items from YCIS and ESQ. Overall, the correlation analysis among measurements provides evidence for the concurrent validity of the SWAN-OM covering the domains for measuring the quality of care (68); further comparison between PANAS at Times 1 and 2 provides further construct validity on affect changes after SST.

The Shapiro–Wilk test of normality confirmed non-normal distribution (significant at *p* < 0.05) routine data collected in digital mental health services may have outliers as well as variability in the completion rates of each questionnaire (e.g., noise and connectivity), so it is expected that the natural data collection procedure from the evaluation sample may not follow a normal distribution despite the volume of participants. Therefore, Spearman-ranked correlations were used for the analysis. Each pairwise correlation was calculated using the data available for each SWAN-OM item (Supplementary Table 7) and each comparator measure. In instances where individuals had completed the SWAN-OM and comparator measures more than once, within-individual average scores were computed prior to calculating the correlations. Pairwise correlations with <20 cases were not reported. Only cases where the SWAN-OM was completed for Time 1 and Time 2 were included, comprising a subsample of 577 young people attending 696 chats that were used for these analyses.

## 3. Results

### 3.1. Completion rates and descriptive statistics

Most young people accessing the service for a SST chat selected SWAN-OM items before their session (SWAN-OM pre-chat item selection; *n* = 1,503, 78.69%). After their chat session, 696 young people completed the measure scoring (46.31%), while 112 (7.45%) users skipped the measure scoring and 695 (46.24%) left the chat before the measure scoring could be presented during the evaluation. When considering only individuals who saw the SWAN-OM and were able to complete their chat intervention, the vast majority completed the measure scoring after their chat session (*N* = 969, 86.14%), providing a good indicator of acceptability and completion rates for the instrument.

Completion rates from the other comparative instruments were high in the pre-chat item selection, with more than 73% of young people selecting items on the measure. After the session, measure engagement rates decreased overall, with <24% of the sample completing the PANAS, YCIS, and ESQ (Supplementary Table 1).

The SWAN-OM allows young people to select up to three items from a list of 21 items by previously selecting between six-theme categories to display a group of items (between 2 and 5), and there is no limitation on the number of themes that young people can select, allowing in that scenario to display up to all 21 items for choice. Young people were selected on average and 2.47 items (SD = 0.76; median = 3).

The frequency of item selection within the overall sample ranged in selection rates from 0.51 to 11.61% (Table 2). The frequency analysis identified popular items among the sample, such as “*Feel better*” (*N* = 431; 11.61%) and “*Find ways I can help myself* ” (*N* = 411; 11.07%), and unpopular items, such as “*Feel safe in my relationships*” (*N* = 53; 1.43%) or “*Learn the steps to achieve something I want*” (*N* = 58; 1.56%), with “*Learn how to relate to other people”* as the least selected item (*N*= 19; 0.51%).

**Table 2:** SWAN-OM item selection frequencies, pre-post completion rates, and item average scores^a^^b^.

**Theme**	***N***	**Item**	**Pre-chat item selection**		**Post-chat item scoring**			
			***n***	**%**	***n***	**%**	**Mean**	**SD**
A		Total	470	12.66%				
	1	Be comfortable asking for help outside Kooth	276	7.43%	134	7.89%	0.54	1.00
	2	Find information about how to keep myself safe	194	5.22%	95	5.59%	1.18	0.87
B		Total	991	26.69%				
	3	Feel listened to	333	8.97%	138	8.13%	1.58	0.71
	4	Talk about something I haven't told anyone before	187	5.04%	71	4.18%	1.32	1.19
	5	Identify a solution to a problem in my life	179	4.82%	75	4.42%	0.96	0.85
	6	Be able to open up to people in my life	169	4.55%	67	3.95%	0.58	0.99
	7	Find out how useful it is to talk to someone	70	1.89%	27	1.59%	1.44	0.85
	8	Feel safe in my relationships	53	1.43%	20	1.18%	0.5	0.95
C		Total	150	4.04%				
	9	Learn how to feel better	92	2.48%	44	2.59%	1.00	1.08
	10	Learn the steps to achieve something I want	58	1.56%	35	2.06%	1.14	1.03
D		Total	967	26.04%				
	11	Understand my feelings and/or behaviors	373	10.05%	159	9.36%	0.72	0.87
	12	Identify ways to help me worry less	233	6.28%	112	6.60%	0.96	0.92
	13	Explore how I feel	225	6.06%	114	6.71%	1.16	1.02
	14	Be more comfortable with my feelings	136	3.66%	64	3.77%	0.98	0.88
E		Total	215	5.79%				
	15	Explore difficulties in my relationships	78	2.10%	37	2.18%	0.78	1.23
	16	Identify solutions to improve my relationships	75	2.02%	38	2.24%	0.76	1.02
	17	Learn how to manage conflict with others	43	1.16%	22	1.30%	1.14	0.77
	18	Learn how to relate to other people	19	0.51%	12	0.71%	0.33	0.89
F		Total	842	22.68%				
	19	Feel better	431	11.61%	212	12.49%	0.90	0.94
	20	Find ways I can help myself	411	11.07%	186	10.95%	1.06	0.96
NA	21	Free text	78	2.10%	36	2.12%	0.94	1.17

a Frequency of SWAN-OM item selection is provided for pre-chat item selection, percentages at Time 1 are calculated based on total (n = 3,713) items selected during 1,503 chats; frequency, mean, and standard deviation (SD) are provided for SWAN-OM items at Time 2, percentages at post-chat item scoring are calculated based on total (n = 1,698) items rated during 696 chats.

b Themes: A, ‘Understand what help I can get'; B, ‘Share my story with someone'; C, ‘Set and achieve my goals'; D, ‘Explore my emotions'; E, ‘Improve my relationships'; F, ‘Learn ways to cope'. N, item number.

### 3.2. Predicting SWAN-OM item selection

There was an acceptable amount of data to compute hierarchical logistic regressions on the five most selected items during the evaluation. For the items “*Feel better*” and “*Identify my feelings or/and behaviors*”, no significant differences between demographic variables were found predicting the selection of these two items (p > 0.05).

Hierarchical logistic regressions for the rest of the items accounted for some differences in item selection prediction across demographic characteristics. The item “*Find ways I can help myself* ” showed that the demographic characteristics of young people significantly predicted the selection of this item (Table 3). Compared to females, males were more likely to select this item (OR = 0.51, CI = [0.3–0.87]; overall model significance: $X_{\left(5\right)}^{2}$ = 8.95, *p* = 0.03). With regard to ethnicity, Black African, Black Caribbean, and Black British individuals were significantly less likely to select the item when compared to white young people (OR = 0.27; CI = [0.08–0.86]; $X_{\left(11\right)}^{2}$ = 22.81, *p* < 0.001); this difference in item selection was also found for people from any other ethnic group and those who did not provide ethnicity data (OR = 0.12, CI = [0.03–0.53]). Asian and Asian British were also less likely to select “*Find ways I can help myself* ” albeit significant at a trend level (OR = 0.56; CI = [0.28–1.11]), as well as those young people experiencing presenting concerns around risk within the service (OR = 0.56, CI = [0.32–0.98]; $X_{\left(16\right)}^{2}$ = 9.26, *p* = 0.10).

**Table 3:** Results of the hierarchical multi-level logistic regressions for SWAN-OM Item 20: “Find ways I can help myself”^a^.

**SWAN-OM Item 20**	**OR**	***p*** **-value**	**95% CI**
**Gender** ^*^			
Male vs. female	0.51^*^	0.013	0.30–0.87
Agender vs. female	0.59	0.229	0.25–1.4
Gender fluid vs. female	1.14	0.752	0.50–2.64
**Age**			
10–14 vs. 15–19	0.79	0.181	0.55–1.12
20 and above vs. 15–19	0.88	0.667	0.50–1.57
**Ethnicity** ^*^			
Asian/Asian British vs. White	*0.56^+^*	0.094	0.28–1.11
Black African/Caribbean/Black British vs. White	0.27^*^	0.027	0.08–0.86
Mixed multiple ethnic group vs. White	0.69	0.256	0.36–1.31
Any other/Unknown vs. White	0.12^*^	0.005	0.03–0.53
**Presenting concerns** ^b^			
Mental health	0.97	0.889	0.65–1.45
External	0.90	0.542	0.66–1.25
Suicidal thoughts/self-harm	1.23	0.211	0.89–1.72
Risk	*0.56^+^*	0.042	0.32–0.98
Physical/other	0.60	0.153	0.30–1.21

a Data collected from 910 young people attending 1,131 chats. OR, odds ratio. CI, confidence interval. For variables to be significant, both the model in which they were entered and the effect of the variable within the model had to meet the p < 0.05 threshold. Model significance is indicated in parenthesis next to the predicting variable. (^*^) indicates a significant effect at p < 0.05. (+) indicates an effect at trend level (p < 0.1).

b Adding presenting concerns did not reach p < 0.05 significant level for model fit; therefore, although the effect of risk was significant, we have interpreted this as trend level.

For the item “*To understand my feelings and/or behaviors*”, we found differences in age and gender in item selection as predictive characteristics significant in the model (Table 4). The results showed that young people aged between 10 and 14 years were significantly less likely to select this item when compared to those aged 15–19 years (OR = 0.58, CI = [0.38–0.88]; $X_{\left(7\right)}^{2}$ = 7.88, *p* = 0.02). Males were also less likely to select this item compared to females, albeit at a trend level (OR = 0.54, CI = [0.30–0.96]; $X_{\left(5\right)}^{2}$ = 5.76, *p* = 0.12). Other characteristics did not show any significant power to predict the selection of this item.

**Table 4:** Results of the hierarchical multi-level logistic regressions for SWAN-OM Item 13: “*Understand my feelings and/or behaviours*”^a^.

**SWAN-OM Item 13**	**OR**	***p*** **-value**	**95% CI**
**Gender** ^+^			
Male vs. female	0.54^+^	0.035	0.30–0.96
Agender vs. female	0.59	0.293	0.22–1.58
Gender fluid vs. female	0.76	0.591	0.29–2.04
**Age** ^*^			
10–14 vs. 15–19	0.58^*^	0.010	0.38–0.88
20 and above vs. 15–19	1.09	0.782	0.58–2.05
**Ethnicity**			
Asian/Asian British vs. White	1.00	1.000	0.48–2.07
Black African/Caribbean/Black British vs. White	0.94	0.916	0.33–2.73
Mixed multiple ethnic group vs. White	0.97	0.933	0.48–1.98
Any other/Unknown vs. White	1.93	0.157	0.78–4.82
**Presenting concerns**			
Mental health	1.17	0.496	0.74–1.84
External	0.90	0.556	0.63–1.29
Suicidal thoughts/self-harm	1.00	0.997	0.69–1.45
Risk	0.63	0.140	0.34–1.16
Physical/other	1.13	0.724	0.57–2.27

a Data collected from 910 young people attending 1,131 chats. OR, odds ratio. CI, confidence interval. For variables to be significant, both the model in which they were entered and the effect of the variable within the model had to meet the p < 0.05 threshold. Model significance is indicated in parenthesis next to the predicting variable. (^*^) indicates a significant effect at p < 0.05. (+) indicates an effect at trend level (p < 0.1).

Finally, for item 6 “*Feel listened to*” from the SWAN-OM, hierarchical regression results indicated that young people experiencing risk as a presenting concern within the service were significantly more likely to select this item (OR = 2.51, [1.28–4.9]; $X_{\left(16\right)}^{2}$ = 11.83, *p* = 0.04) compared to those without risk issues. A trend difference in age was found in those between 10 and 14 years who were more likely to select this item compared to those aged 15–19 years (OR = 1.59, CI = [1.03–2.45]; $X_{\left(7\right)}^{2}$ = 4.95, *p* = 0.08) (Table 5).

**Table 5:** Results of the hierarchical multi-level logistic regressions for SWAN-OM Item 6: “*Feel listened to*”^a^.

**SWAN-OM Item 6**	**OR**	***p*** **-value**	**95% CI**
**Gender**			
Male vs. female	0.71	0.267	0.38–1.31
Agender vs. female	1.43	0.466	0.55–3.7
Gender fluid vs. female	1.62	0.342	0.6–4.42
**Age** ^+^			
10–14 vs. 15–19	*1.59^+^*	0.037	1.03–2.45
20 and above vs. 15–19	0.86	0.690	0.41–1.79
**Ethnicity**			
Asian/Asian British vs. White	1.57	0.251	0.73–3.40
Black African/Caribbean/Black British vs. White	2.00	0.228	0.65–6.16
Mixed multiple ethnic group vs. White	0.95	0.888	0.43–2.06
Any other/Unknown vs. White	0.90	0.840	0.33–2.49
**Presenting concerns** ^*^			
Mental health	1.01	0.958	0.63–1.64
External	1.26	0.261	0.84–1.89
Suicidal thoughts/self-harm	0.93	0.709	0.61–1.39
Risk	2.51^*^	0.007	1.28–4.90
Physical/other	1.64	0.199	0.77–3.48

a Data collected from 910 young people attending 1,131 chats. OR, odds ratio. CI, confidence interval. For variables to be significant, both the model in which they were entered and the effect of the variable within the model had to meet the p < 0.05 threshold. Model significance is indicated in parenthesis next to the predicting variable. (^*^) indicates a significant effect at p < 0.05. (+) indicates an effect at trend level (p < 0.1).

For the rest of the items of the SWAN-OM predictive analysis, chi-square tests were computed for each item and a single demographic variable. These analyses did not consider the repeated measures within the sample of young people and the co-variance between demographic characteristics. For presenting concerns, comparisons between groups were calculated, i.e., between those with the presenting concerns and those who did not present that problem in the sample.

The chi-square comparisons on presenting concerns (Supplementary Table 2) found that young people who experience presenting concerns around risk (e.g., the victims of crime and trauma) were more likely to select the item “*Feel safe in my relationships*” [$X_{\left(1\right)}^{2}$ = 11.33, *p* < 0.001]. Young people experiencing suicidal thoughts and/or self-harm were less likely to select the item “*Identify solutions to improve my relationships*” [*X*^2^_(1)_ = 11.47 *p* < 0.001] and more likely to select “*Find information about how to keep myself safe”* [$X_{\left(1\right)}^{2}$ = 18.62, *p* < 0.001].

Regarding the chi-square comparisons computed for the rest of the demographic variables, it was found that young people aged 10–14 years were significantly more likely to select the items “*Be able to open up to people in my life*” [$X_{\left(2\right)}^{2}$ = 17.98, *p* < 0.001] and “*Talk about something I haven't told anyone before*” [$X_{\left(2\right)}^{2}$ = 14.67, *p* < 0.001]. The oldest age group (20 years and above) was more likely to select the item “*Identify solutions to improve my relationships*” [$X_{\left(2\right)}^{2}$ = 22.77, *p* < 0.001].

In terms of ethnicity, there appeared to be an effect of young people from Black African/Caribbean and Black British backgrounds being significantly more likely to select the item “*Learn how to relate to other people”* [very small; $X_{\left(4\right)}^{2}$ = 22.30, *p* < 0.001], although a very small cell size indicates that this finding is unreliable. Young people who did not provide ethnicity information and those who identified as part of any other ethnic background were less likely to select the item “*Find ways I can help myself* ” [$X_{\left(4\right)}^{2}$ = 21.18, *p* < 0.001]. No significant differences in item selection for gender were found (Supplementary Table 3).

### 3.3. Variability in SWAN-OM scores

The variability of item scoring was explored by looking at the population average of the selected items at the post-chat item scoring stage when the SWAN-OM was considered completed after a single-session intervention.

Average scores of SWAN-OM items at the post-chat item scoring stage ranged between 0.33 (SD = 0.89) for the item “*Learn how to relate to other people*”, and 1.58 (SD = 0.71) for the item “*Feel listened to*” (Table 2). Some items had larger positive scores on average, perhaps because they captured goals that could be more easily implemented during a single-chat intervention as part of the “in-session” goals within the online service. For instance, “*Feel listened to*” and “*Find out how useful it is to talk to someone*” were among the best-scored items. Other items, such as “*Be able to open up to people in my life*”, “*Feel better*”, or “*Explore difficulties in my relationships*” had on average lower scores across the sample. The interpretation of these scores needs to account for the variation in the number of cases used to calculate the average scores, which varied from 12 to 212 chats for the available 20 items.

### 3.4. Concurrent validity

Pairwise Spearman-ranked correlations were calculated for individual items and total scores for the YCIS, ESQ, and PANAS to explore the hypothesis of how these instruments correlated with the SWAN-OM. Pairwise correlations with <20 cases were not reported. Some of the results (shown in Table 6) should take into consideration the large number of significance tests calculated and the relatively small number of paired cases available for some of the correlation tests (Supplementary Table 4).

**Table 6:** Pairwise Spearman-ranked correlations between individual SWAN-OM items and YCIS, ESQ, and PANAS total scores^a^−^c^.

**N**	**Item**	**ESQ**	**YCIS**	**PANAS-NA**	**PANAS-PA**
1	Be comfortable asking for help outside Kooth	0.413 *p =* 0.001	0.495 *p < * 0.001	−0.186 *p =* 0.113	0.356 *p =* 0.002
2	Find information about how to keep myself safe	0.306 *p =* 0.017	0.452 *p < * 0.001	−0.082 *p =* 0.504	0.239 *p =* 0.049
3	Feel safe in my relationships	–	–	–	–
4	Be able to open up to people in my life	0.219 *p =* 0.199	0.643 *p < * 0.001	−0.199 *p =* 0.200	0.469 *p =* 0.002
5	Talk about something I haven't told anyone before	0.153 *p =* 0.379	0.339 *p =* 0.023	−0.338 *p =* 0.023	0.35 *p =* 0.018
6	Feel listened to	0.329 *p =* 0.005	0.473 *p < * 0.001	−0.238 *p =* 0.031	0.309 *p =* 0.005
7	Find out how useful it is to talk to someone	–	–	–	–
8	Identify a solution to a problem in my life	0.349 *p =* 0.025	0.572 *p < * 0.001	−0.151 *p =* 0.310	0.412 *p =* 0.004
9	Learn how to feel better	0.284 *p =* 0.212	0.595 *p =* 0.002	−0.367 *p =* 0.078	0.717 *p < * 0.001
10	Learn the steps to achieve something I want	–	0.764 *p < * 0.001	−0.215 *p =* 0.313	0.273 *p =* 0.197
11	Explore how I feel	0.255 *p =* 0.060	0.686 *p < * 0.001	−0.445 *p < * 0.001	0.372 *p =* 0.001
12	Be more comfortable with my feelings	0.116 *p =* 0.55	0.634 *p < * 0.001	−0.105 *p =* 0.562	0.372 *p =* 0.033
13	Understand my feelings and/or behaviours	0.388 *p < * 0.001	0.614 *p < * 0.001	−0.389 *p < * 0.001	0.35 *p =* 0.001
14	Identify ways to help me worry less	0.266 *p =* 0.045	0.681 *p < * 0.001	−0.239 *p =* 0.053	0.393 *p =* 0.001
15	Explore difficulties in my relationships	–	0.496 *p =* 0.014	−0.180 *p =* 0.400	0.305 *p =* 0.147
16	Learn how to relate to other people	–	–	–	–
17	Learn how to manage conflict with others	–	–	–	–
18	Identify solutions to improve my relationships	–	0.507 *p =* 0.023	0.004 *p =* 0.987	0.324 *p =* 0.164
19	Feel better	0.483 *p < * 0.001	0.684 *p < * 0.001	−0.301 *p < * 0.001	0.441 *p < * 0.001
20	Find ways I can help myself	0.423 *p < * 0.001	0.599 *p < * 0.001	−0.301 *p =* 0.001	0.423 *p < * 0.001
21	Free text	0.483 *p =* 0.023	0.700 *p < * 0.001	−0.380 *p =* 0.067	0.609 *p =* 0.002

a Items with < 20 cases were not reported. Pairwise correlations are based on varying subsamples depending on the data available.

^b^N, SWAN-OM item number.

c More information is available in Supplementary Table 4.

Analysis revealed that nine SWAN-OM items were positively correlated with the ESQ total scores. The items “*Be comfortable asking help outside Kooth*” [rs_(64)_ = 0.41, *p* < 0.001], “*Understand my feeling and/or behaviors*” [rs_(86)_ = 0.39, *p* < 0.001], “*Feel better*” [rs_(109)_ = 0.48, *p* < 0.001], and “*Find ways I can help myself* ” [rs_(98)_ = 0.42, *p* < 0.001] showed a significant positive association with ESQ total scores at *p* < 0.001. In addition, five items from the SWAN-OM showed a significant positive association with a *p* < 0.05 significance level on ESQ total scores.

A total of 13 items from the SWAN-OM showed a significant positive correlation with the total scores of the YCIS insight subscale. Items such as “*Learn the steps to achieve something I want*” [rs_(22)_ = 0.76, *p* < 0.001], the personalized “*free-text*” option [rs_(22)_ = 0.7, *p* < 0.001], and “*Explore how I feel*” [rs_(70)_ = 0.69, *p* < 0.001] were among the ones with the highest Spearman-rank coefficients showing large associations. Furthermore, additional four items from the SWAN-OM were correlated positively with YCIS total scores at a *p* < 0.05 significance level.

Pairwise correlations were also computed between individual SWAN-OM items and individual items of the YCIS and ESQ. Similar trends were observed between correlations of total scores and individual items from the ESQ and YCIS (Supplementary Tables 5, 6).

Six items from the SWAN-OM showed a significant negative correlation with PANAS Negative Affect subscale (PANAS-NA) total scores. Items such as “*Explore how I feel*” [rs_(70)_ = −0.44, *p* < 0.001] and “*Feel listened to*” [rs_(80)_ = −0.24, *p* < 0.05] were among the higher and lower significant negative correlations. A total of 14 items from the SWAN-OM showed statistically significant correlations with PANAS Positive Affect (PANAS-PA) subscale total scores. The item “*Learn how to feel better*” [rs_(22)_ = 0.72, *p* < 0.001] showed the highest significant association, and “*Find information about how to keep myself safe*” (rs_(66)_ = 0.24, *p* < 0.05) indicated the lowest coefficient among the items showing an association.

Young people experienced a significant improvement in positive affect following their SST, represented by changes in the scores before and after the session in PANAS, with an average change of M = 3.08 (SD = 3.95) between Time 1 and Time 2, *t*_(452)_ = 16.58, (*p* < 0.001). They also experienced a significant reduction in negative affect following their chat, with an average change of *M* = −4.03 (SD = 3.61), *t*_(452)_ = −23.71, (*p* < 0.001). Positive affect significantly changed between pre- and post-SST on average total scores. Negative affect was significantly reduced after SST when comparing pre- and post-scores of the PANAS.

## 4. Discussion

In the context of brief psychotherapy and solution-focused interventions, there is a measurement deficit in demonstrating outcomes within the single-session therapeutic intervention. Evidence suggests that this type of intervention and service delivery can be effective in reducing waiting times and increasing access to psychotherapy and mental health support (38), but the literature examining what factors contribute to SST clinical effectiveness is inconclusive and complex (36, 69). Despite this, evidence on the effectiveness and support for single-session mental health interventions continues to grow (1–4, 16–18). Further studies stress the importance of how a single point of engagement with mental health services appears as a frequent option when monitoring service engagement (70), including 46% of CYP engaging with mental health services in the UK with only one appointment (27); note that this percentage will include treatment drop-outs or people who booked further appointments and were not planned as an SST.

Currently, despite research on outcome measures and examining the effectiveness of single-session intervention continues, even at a more rapid pace in the context of the COVID-19 pandemic and the new mental health needs emerging in the population (2), most of these efforts target specific mental health difficulties (29–31), and none of the previously used instruments have been designed with SST and a pluralistic view of service delivery in mind. There is a fundamental challenge related to the short-term nature of SST tudies and the disconnect with the measurement of mental health difficulties, which are often measured over several time points, or across longer time periods, or where SST outcome measures have been developed: they tend to be solely symptom-based (1, 32).

This article explored the validity of a new outcome measure for SST (the SWAN-OM), which aims to provide a patient-reported outcome measure that captures the “Wants” and “Needs” of the single-session itself and the associated achievement of these wants. This article focused on examining concurrent and convergent validity, as well as importantly exploring how the measure is used by different individuals, by studying demographic characteristics and item selection. By providing early evidence for instrument validation of the SWAN-OM, we hope that this measurement may also contribute to demonstrating the effectiveness of SST across services.

The novel design provides young people with the most approachable version of the instrument, given that the structural design was driven by young people's participatory design ideas (50). This is novel and exciting, as it puts the user at the forefront of the instrument's design. While the structure of the SWAN-OM measures means that traditional psychometric testing may not be appropriate, this offers the opportunity to examine this measure at an item level. In addition, some considerations should be taken for psychometric evaluation and exploration of properties, particularly when attempting to provide test-retest reliability. Test-retest reliability is not applicable to the SWAN-OM instrument; no two SSTs are the same experience, as it is not reasonable to examine the repeated measurement of SST outcomes, given that the main SST principle is rooted in the possibility that the intervention will be the “one and only” encounter between the practitioner and client (3). An advantage of this novel instrument use is that the SWAN-OM changes to fit the needs of the young person for every SST they attend and was designed for a pluralistic workforce and approach to counseling. The SST outcomes are expected to change from one session to the next one; hence, the SWAN-OM changes, too, with each administration, so each SWAN-OM is personalized to each SST and the interactions between practitioner and user, focusing the measurement on the intervention impact rather than the information of the underlying factor structure that contributes to that impact from a measurement point of view. Despite this, there have been efforts to conceptualize these non-specific factors on SST (42, 71) and some of them may be related to basic psychological needs used to build the statement of the instrument. Overall, future advances in SST and psychotherapy research should continue to investigate and unveil those factors, as well as explore an empirically grounded and systematic taxonomy of activities that are known to be more therapeutic (e.g., listening, alliance, and foster autonomy) in these brief but potentially important encounters with professionals and support (64); this would ensure the effectiveness of SST can continue to improve alongside its popularity, scalability, and impact to provide wellbeing to society through professional support and help with therapy.

### 4.1. Principal results

#### 4.1.1. SWAN-OM evaluation completion rates

Completion rates are of high importance when examining a new outcome measure. The completion rates of the SWAN-OM at pre-chat item selection was high, with over three-quarters (78.69%) of the young people who saw the measure completing it. Of the young people who viewed the post-session SWAN-OM, there was a high completion rate, showing good acceptability of the measure (86.14%). This suggests that young people found the measure reasonable to complete after an SST and is in line with previous research, which showed the SWAN-OM had good levels of acceptability and face validity (50).

This is encouraging, as the two-stage structural design of the SWAN-OM was created as a way to organize information (72, 73) and reduce cognitive load (74) on the young people completing the instrument. In addition, from this evaluation, it appears that three items are enough to take forward to the session, as most young people selected between two and three items. This is in line with what practitioners said during the instrument development (50); three goals were a manageable number of “in-session” objectives and set out clear expectations about what can be achieved in a SST encounter between practitioner and CYP requesting support.

#### 4.1.2. SWAN-OM item selection and variability

On an item level, age, ethnicity, and gender significantly predicted differences in some of the item-level selections by young people. For instance, young people who had risk-related presenting concerns were more likely to select the items “*Feel listened to*”, highlighting the importance of distress disclosure (75), and “*Feel safe in my relationships*”, which is in line with the presenting concerns risk grouping often covering abuse, trauma, and bullying. It is important to note that the instrument was never designed to identify or assess risk, and, while the evaluation took place, any risk disclosure overruled the completion of the SWAN-OM and the activities of SST and protocol to work with the instrument responses and instead became a safeguarding and crisis support within the service, as per clinical governance, providing different activities within the SST to ensure no harm and the safety of the participant (e.g., routine inquiry about suicide risk, standardized assessment, and crisis action plan development). This may have, in turn, affected some of the results and scores in the instrument during the evaluation.

Overall, despite differences seen at the item level through exploring demographic characteristics, the SWAN-OM was designed for and administered to a wide range of ages and backgrounds (Kooth is accessible to users aged 11–25 years), with choice and a person-centered approach at the heart of the measure design. Therefore, the standardization in the selection of all items was unlikely to be achieved across items; yet we recommend further investigation to determine if the instrument should be adapted based on specific characteristics such as age and cultural factors, especially for future cross-cultural validation studies (76). The current evidence suggests that some differences in culture, age, or gender may affect the performance of the instrument when selecting items but demonstrates no differences in the two most frequently selected items of the SWAN-OM; a larger sample size with more items selected is recommended to test this predictive approach to each item of the instrument. These findings provide some indication about potential cultural adaptions that the instrument may require to undergo to standardize and serve underrepresented and minority populations within a digital mental health platform with universal access.

Regarding the variability of scores for items, some SWAN-OM items, when selected, were scored lower on average than others; however, the overall average of “in-session” pre-defined goal scores was positive across all items. This highlights the importance of specificity in measurement when dealing with change in an SST intervention so that change can be directly reported after the intervention. The SWAN-OM scores the perception of change in individual “in-session” goals for the single-session, despite non-specific factor effects on practitioner skills, therapeutic alliance, and contextual factors of the session (77). The assumption from these results is that single-session outcomes included in the SWAN-OM can be achieved in the context of SST. However, differences in average scores were found between items, leading to the question of whether certain items are realistically achievable in one session, e.g., “*Feel better*”, which suggests a change and maintenance of a positive emotional state of the individual and may be unrealistic from the input of one session, or may not change when dealing with negative affect responses in psychotherapy (78). In addition, “*Explore difficulties in my relationship*” can be particularly difficult to address in one session alone, as interpersonal functioning can be difficult to describe or understand, with evidence that long periods of therapeutic input are required for clients to report changes in interpersonal functioning (79–81). These examples provide some indication for item modification or deletion and suggest further research to collect feedback from practitioners and CYP about the use of the SWAN-OM, and they also provide an initial indication of how personal characteristics may influence the difficulty score in each item of the measure.

#### 4.1.3. SWAN-OM concurrent and construct validity

Three different instruments and subscales were used to explore how well the measure interacts with other well-established standardized measures. Overall, we observed good concurrent validity between individual SWAN-OM items and the comparator measures (PANAS, YCIS, and ESQ), meaning that some, but not all, items were associated with negative and positive affect, experience or satisfaction, and impact of the session. The exploration between PANAS at Times 1 and 2 showed a significant change in positive affect and a reduction of negative affect immediately after the single session; this may be linked with the often reported high rates of satisfaction levels in SST and walk-in clinic studies (3, 24, 82) and highlights that affect changes are likely to occur as a result of an SST intervention (4). This is an important finding that may indicate construct validity for the instrument and the intervention, as well as provide evidence on the effectiveness and changes that take place after the one and only encounter of therapy. Further exploration of the constructs that the measure is intending to capture is required and may be linked to agency, hope, and other psychological constructs associated with single-session interventions (2, 28).

### 4.2. Study limitations and wider considerations

There are wider considerations when examining the findings regarding the psychometric properties of the SWAN-OM. Some of these considerations are commonly encountered in psychometric testing research; others are related to the idiographic nature of one of the items, as well as the dynamic nature of the assessment, time of observation for intervention; and other common challenges like sample size and population diversity are also found in the applied research context (83). The nature of the measure focuses on measuring the SST intervention; therefore, the changes in scores will not be expected to be maintained across time when administered twice. Therefore, the psychometric consistency of SWAN-OM scoring could not be calculated through common test-retest approaches. Furthermore, the item selection process in the instrument defines the construct of the SST, making internal consistency values and their interpretation less relevant (84). Item selection was purposefully limited to young people being able to select between one and three items, impacting the volume of data per item. This limitation by design was determined by clinical judgment based on the expected number of objectives that can be covered in SST. Moreover, the pairwise comparisons and correlations often had low sample sizes for certain items that were selected less commonly during the audit. Yet, inter-item correlations were explored for those items with enough responses, showing overall significant correlations between items in line with the reliability scores of themes. Regarding items to focus on in the single session, some preliminary exploration of the reliability of items for the SWAN-OM has been examined by experts through content validity indexes during the instrument development process (50).

Taking this into consideration, the SWAN-OM is recommended to be used at the item level where each item is treated individually as part of SST, rather than at the theme level. This provides the granularity for practitioners and young people to match up the “Wants” and “Needs” with session progress, experience, and structure. Items can be aggregated across a young person's session, or across specific items between young people, to monitor SST appropriateness in supporting that specific “Want” or “Need”. Further investigation of practitioner perceptions of total scores, multiple administration scores, or even service-level data usability for each item and the total score of the instrument is required. The SWAN-OM does not rate the perception of the clinician on what outcomes were achieved, who is suggested to be the best stakeholder to discern the effectiveness and impact of SST to date. In contrast, the SWAN-OM is the first ever patient-reported outcome measure designed for SST in a digital environment; further studies should look at how the clinician-reported and patient-reported measures interact when measuring SST. Finally, this evaluation was conducted at one digital web-based mental health service, as part of service improvement, therefore, restricting the generalizability of the results to other digital contexts or non-text-based SSTs. This is important, as the instrument was also developed through a service-specific program theory, making it difficult to interpret and define the individual constructs being measured (85) and ensure the items are related to wider services and represent the same common “Wants” and “Needs” from SST across CYP. The results provided in this study suggest that characteristics such as age and ethnicity can predict item selection, literacy, and comprehension, and other factors may contribute to these differences as well as indicate a potential need for adaptation of certain items to better serve the 11–25 year-old population. The results also show some indication of questionnaire fatigue in line with previous studies (86), and considerations should be made for balancing the number of instruments that can be used for comparisons between instruments when testing validity, especially in the digital context. Future research should explore response rates and item selection preferences in non-digital and non-text-based services.

### 4.3. Future research

Future research on the instrument's ongoing validity should try to answer what works best for whom and under what conditions, in an attempt to generalize its use and standardization. This is especially important due to the wide variability of practices that someone may observe in single-session therapeutic encounters. Sequential and optimization trials may help to determine the branching logic effectiveness or suggest better ways to adapt the questionnaire to the characteristics of the individual, and further studies on demographic differences and statement comprehension and understanding may provide different versions and adaptations of the instrument.

We also find some items that performed poorly or were rarely selected, suggesting some future item changes toward an optimized tool or simply adapting the current set of statements. Therefore, we recommend making some items less absolute, as these items were selected less frequently and appear to be harder to achieve in a single session, and hence they are being selected less frequently. An example of this would be changing the item “*Feel better*” to a less absolute version, perhaps more grounded on temporality and immediacy: “*To start to feel better*”. This is more in line with the outcomes that can be achieved in a single session, and we expect this to result in a more equal spread of item selection. Therefore, future research needs to examine the inclusion of certain items or adaptations to the instrument. We recommend input from stakeholder groups, especially to examine the validity of the instrument in other walk-in services provided in the community.

Finally, with the increased need and popularity of SSTs, we recommend exploring the perception of meaningful change after a single-session and how this aligns with SWAN-OM outcome scores, and practitioners' perceptions of the scores may help to determine a threshold for significant change behind the scale. A longitudinal-controlled study could then examine the maintenance and magnitude of change in SST for those who were administered with the SWAN-OM in their sessions. This will also provide evidence of how such walk-ins and SST service delivery can impact, at a large scale, waiting times, and access across the wider mental health welfare system (38). Many consider SST as an intervention that lay professionals can deliver and master, and this could benefit to address the shortage and build effective support mechanisms in society in order to provide support at the time of need for those who are seeking help. However, further trials are needed to understand the cost-effectiveness of this type of therapeutic approach for digital and non-digital contexts, and perhaps its interaction in a health system.

## 5. Conclusion

In this article, we presented evidence on the validity of a new outcome measure for SST (the SWAN-OM), which is a patient-reported outcome measure that captures the “Wants” and “Needs” of the SST itself. The outcome and experience aspects measure the associated achievement of these “Wants” or “Needs” and the experiences of the session from the perspective of the client (CYP: 11–25 years-old).

This evaluation showed positive results on some of the psychometric properties assessed when the data and the context-applied methodological design allowed it. Wider considerations have been discussed on the novel structure and two-stage logic of the measure. Nevertheless, there are indications of good construct validity by using and comparing concurrent instruments that showed good reliability while evaluated, and further verification that changes in affect scores take place after the SST with differences found between the pre-chat and post-chat measurement during the auditing period.

Our evaluation methods and findings provide a way to compare and validate measures that may be required of a specific design and structure, and acknowledge the challenges ahead to align new technological advancements in questionnaire development and personalization with psychometric properties and validation. This opens a door to the proliferation of measures that consist of a combination of nomothetic and idiographic items (49, 87). Outcome and experience measurement will continue to proliferate in digital contexts; it is important that advances are made to assess the properties of these instruments and how they may be used outside the digital context, and their validity in face-to-face mental health services, so innovations in digital mental health support may be of use when implemented in mainstream practices, as well as contribute to the knowledge and evidence-based SST.

## Data availability statement

The raw data supporting the conclusions of this article will be made available by the authors, without undue reservation. The datasets generated during and/or analyzed during the current evaluation study are available from the corresponding author at research@kooth.com. Datasets are not publicly available due to the privacy and consent of the service users whose data were used.

## Ethics statement

Ethical review and approval was not required for the study on human participants in accordance with the local legislation and institutional requirements. Written informed consent from the participants' legal guardian/next of kin was not required to participate in this study in accordance with the national legislation and the institutional requirements.

## Author contributions

SDOG is the main author and led the conception and design of the study and measure. AS coordinated the evaluation at Kooth, wrote sections of the manuscript, and responsible for supervision and project administration. LS supported the implementation within Kooth mental health digital service. JJ and JE-C contributed in the design and evaluation of the study as advisors at CORC and influenced the original design of the measure, contributed to use of the comparator measures of the study, advised on data extraction, led the analysis plan, and had oversight of the analysis. SDOG and LS led the implementation of the measures and data collection for the study and organized the database. SDOG, LS, and AS trained practitioners on the procedure and measure. FR ran the analysis and contributed to the manuscript analysis plan. SDOG, LS, JE-C, and JJ provided most of the contributions to the manuscript. All authors contributed to the manuscript revision, read, and approved the submitted version.
