# Supplementary material for: Examining concurrent validity and item selection of the Session Wants and Needs Outcome Measure (SWAN-OM) in a children and young people web-based therapy service
Source: Front Psychiatry. 2023 Feb 9;14:1067378. doi: 10.3389/fpsyt.2023.1067378 (PMC9947788; doi:10.3389/fpsyt.2023.1067378)
Supplement: Supplementary file 1 [file Table_1.docx]

Supplementary Material

1. **Supplementary Tables**

**Supplementary Table 1.** Completion rates for all outcome measures. ^a b^

|  | Pre-chat stage | | Post-chat stage | | Paired outcome rate | |
| --- | --- | --- | --- | --- | --- | --- |
|  | n | % | n | % | n | % |
| SWAN-OM | 1503 | 78.69 | 701 | 36.70% | 696 | 46.31% |
| PANAS NA | 1407 | 73.66 | 453 | 23.72% | 453 | 32.20% |
| PANAS PA | 1401 | 73.35 | 453 | 23.72% | 453 | 32.33% |
| YCIS | NA | NA | 453 | 23.72% | NA | NA |
| ESQ | NA | NA | 383 | 20.05% | NA | NA |

^a^ *The total sample included data collected from 1401 young people attending 1910 chats.*

^b^ *NA: Not Applicable.*

**Supplementary Table 2.** Frequency of item selection and Chi-square comparisons depending on sample presenting concerns. ^a-d^

|  |  | MH | | Ext |  | SS |  | R |  | PO |  |
| --- | --- | --- | --- | --- | --- | --- | --- | --- | --- | --- | --- |
| N | Item | No | Yes | No | Yes | No | Yes | No | Yes | No | Yes |
| 1 | Be comfortable asking for help outside Kooth | 44 | 169 | 116 | 97 | 123 | 90 | 198 | 15 | 197 | 16 |
| 2 | Find information about how to keep myself safe | 46 | 105 | 81 | 70 | 63- | 88* | 139 | 12 | 138 | 13 |
| 3 | Feel safe in my relationships | 12 | 27 | 13 | 26 | 24 | 15 | 28 | **11*** | 35 | 4 |
| 4 | Be able to open up to people in my life | 27 | 97 | 57 | 67 | 70 | 54 | 106 | 18 | 115 | 9 |
| 5 | Talk about something I haven't told anyone before | 43 | 92 | 61 | 74 | 72 | 63 | 112 | 23 | 123 | 12 |
| 6 | Feel listened to | 66 | 198 | 123 | 141 | 158 | 106 | 223 | 41 | 244 | 20 |
| 7 | Find out how useful it is to talk to someone | 10 | 45 | 32 | 23 | 35 | 20 | 51 | 4 | 49 | 6 |
| 8 | Identify a solution to a problem in my life | 27 | 106 | 61 | 72 | 89 | 44 | 118 | 15 | 126 | 7 |
| 9 | Learn how to feel better | 9 | 56 | 31 | 34 | 40 | 25 | 62 | 3 | 61 | 4 |
| 10 | Learn the steps to achieve something I want | 9 | 36 | 27 | 18 | 30 | 15 | 41 | 4 | 43 | - |
| 11 | Explore how I feel | 37 | 128 | 78 | 87 | 94 | 71 | 146 | 19 | 155 | 10 |
| 12 | Be more comfortable with my feelings | 22 | 86 | 54 | 54 | 62 | 46 | 99 | 9 | 105 | 3 |
| 13 | Understand my feelings and/or behaviours | 57 | 239 | 148 | 148 | 172 | 124 | 272 | 24 | 277 | 19 |
| 14 | Identify ways to help me worry less | 39 | 127 | 77 | 89 | 99 | 67 | 151 | 15 | 157 | 9 |
| 15 | Explore difficulties in my relationships | 22 | 41 | 19 | 44 | 41 | 22 | 56 | 7 | 62 | - |
| 16 | Learn how to relate to other people | 5 | 7 | - | 10 | 10 | - | 12 | - | 12 | - |
| 17 | Learn how to manage conflict with others | 7 | 27 | 14 | 20 | 21 | 13 | 30 | 4 | 30 | 4 |
| 18 | Identify solutions to improve my relationships | 15 | 45 | 18 | 42 | 48* | 12- | 54 | 6 | 57 | 3 |
| 19 | Feel better | 65 | 256 | 159 | 162 | 198 | 123 | 291 | 30 | 308 | 13 |
| 20 | Find ways I can help myself | 70 | 247 | 160 | 157 | 169 | 148 | 295 | 22 | 303 | 14 |
| 21 | Free text | 12 | 40 | 32 | 20 | 24 | 28 | 44 | 8 | 49 | 3 |

^a^ *Data collected from 910 young people attending 1131 chats.*

^b^ *N: SWAN-OM item number; MH: mental health issues; Ext: External issues; SS: Suicidal thoughts / self-harm; R: Risk; PO: Physical / Other issues.*

^c^ *Significant Chi-square tests at p < .001 are denoted as ‘*’ for a higher number and ‘-‘ for a lower number of observed cases in the cell compared to the expected distribution.*

^d^ *Cells with frequencies <3 were suppressed*

**Supplementary Table 3.** Frequency of item selection and Chi-square comparisons separated depending on Gender, Age and Ethnicity. ^a b c^

|  | Gender | | | | Age | | | Ethnicity | | | | | |
| --- | --- | --- | --- | --- | --- | --- | --- | --- | --- | --- | --- | --- | --- |
| Item | F | M | Ag | Gf | 15-19 | 10-14 | 20+ | Wh | As | Bl | Mi | Un |  |
| 1 | 175 | 23 | 12 | 3 | 124 | 74 | 15 | 170 | 13 | 7 | 17 | 6 |  |
| 2 | 132 | 10 | 3 | 6 | 87 | 50 | 14 | 119 | 10 | 5 | 12 | 5 |  |
| 3 | 33 | 3 | - | - | 27 | 11 | - | 29 | 4 | - | 4 | - |  |
| 4 | 96 | 13 | 9 | 6 | 60 | 58* | 6 | 98 | 11 | 3 | 5 | 7 |  |
| 5 | 102 | 15 | 9 | 9 | 68 | 60* | 7 | 107 | 8 | 6 | 11 | 3 |  |
| 6 | 211 | 26 | 14 | 13 | 147 | 99 | 18 | 205 | 21 | 11 | 17 | 10 |  |
| 7 | 45 | 7 | 3 | - | 32 | 20 | 3 | 40 | 4 | - | 3 | 6 |  |
| 8 | 105 | 17 | 6 | 5 | 84 | 37 | 12 | 102 | 12 | 6 | 6 | 7 |  |
| 9 | 56 | 5 | 4 | - | 44 | 18 | 3 | 52 | 4 | 3 | 4 | - |  |
| 10 | 37 | 6 | - | - | 25 | 16 | 4 | 32 | - | 3 | 7 | - |  |
| 11 | 136 | 21 | 4 | 4 | 111 | 37 | 17 | 134 | 10 | 4 | 11 | 6 |  |
| 12 | 92 | 10 | 4 | - | 60 | 39 | 9 | 86 | 5 | 4 | 9 | 4 |  |
| 13 | 253 | 26 | 8 | 9 | 194 | 73 | 29 | 239 | 18 | 8 | 18 | 13 |  |
| 14 | 138 | 17 | 7 | 4 | 109 | 42 | 15 | 139 | 10 | 5 | 5 | 7 |  |
| 15 | 42 | 16 | - | 4 | 39 | 13 | 11 | 48 | 7 | 3 | 3 | - |  |
| 16 | 10 | - | - | - | 8 | 4 | - | 9 | - | 3* | - | - |  |
| 17 | 24 | 8 | - | - | 22 | 9 | 3 | 29 | - | - | - | - |  |
| 18 | 46 | 12 | - | - | 34 | 11 | 15* | 49 | - | 3 | 5 | - |  |
| 19 | 259 | 42 | 11 | 9 | 204 | 88 | 29 | 269 | 16 | 6 | 23 | 7 |  |
| 20 | 271 | 25 | 9 | 12 | 207 | 85 | 25 | 280 | 14 | 4 | 17 | 2- |  |
| 21 | 42 | 6 | - | 3 | 26 | 22 | 4 | 42 | 3 | - | 4 | - |  |

^a^ *Data collected from 910 young people attending 1131 chats. Significant Chi-square tests at p < .001 are denoted as ‘*’ for a higher number and ‘-‘ for a lower number of observed cases in the cell compared to the expected distribution.*

^b^ *N: SWAN-OM item number; F: female; M: male; A: agender; Gf: gender fluid. Wh: White; As: Asian/Asian British; Bl: Black African / Caribbean/ Black British; Mi: Mixed multiple ethnic group; Un: Any other /Unknown.*

^c^ *Cells with frequencies <3 were suppressed*

**Supplementary Table 4.** Pairwise correlation sub-samples. ^a^

| Item | ESQ | YCIS | PANAS-NA | PANAS-PA |
| --- | --- | --- | --- | --- |
| 1 | 66 | 74 | 74 | 74 |
| 2 | 61 | 68 | 68 | 68 |
| 3 | 11 | 13 | 13 | 13 |
| 4 | 36 | 43 | 43 | 43 |
| 5 | 35 | 45 | 45 | 45 |
| 6 | 70 | 82 | 82 | 82 |
| 7 | 13 | 18 | 18 | 18 |
| 8 | 41 | 47 | 47 | 47 |
| 9 | 21 | 24 | 24 | 24 |
| 10 | 18 | 24 | 24 | 24 |
| 11 | 55 | 72 | 72 | 72 |
| 12 | 29 | 33 | 33 | 33 |
| 13 | 80 | 89 | 89 | 89 |
| 14 | 57 | 66 | 66 | 66 |
| 15 | 18 | 24 | 24 | 24 |
| 16 | 7 | 9 | 9 | 9 |
| 17 | 10 | 12 | 12 | 12 |
| 18 | 15 | 20 | 20 | 20 |
| 19 | 111 | 136 | 136 | 136 |
| 20 | 100 | 116 | 116 | 116 |
| 21 | 22 | 24 | 24 | 24 |

^a^ *Data based on an initial sample of 577 young people who attended 696 chats and completed the SWAN-OM at both the pre-chat stage and post-chat stage.*

^b^ *Themes: A: ‘Understand what help I can get’; B: ‘Share my story with someone’; C: ‘Set and achieve my goals’; D: ‘Explore my emotions’; E: ‘Improve my relationships’; F: ‘Learn ways to cope’. N: item number.*

**Supplementary Table 5.** Pairwise correlations between individual SWAN-OM items and individual ESQ. ^a-c^

|  | ESQ |  |  |  |  |  |  |  |  |
| --- | --- | --- | --- | --- | --- | --- | --- | --- | --- |
| ID | 1 | 2 | 3 | 4 | 5 | 6 | 7 | 8 | 9 |
| 1 | 0.267 p=.023* | 0.302 p=.009* | 0.281 p=.016* | 0.266 p=.023* | 0.266 p=.024* | 0.376 p=.001*** | 0.281 p=.018* | 0.318 p=.006* | 0.237 p=.042* |
| 2 | 0.487 p<.001*** | 0.277 p=.025* | 0.289 p=.017* | 0.38 p=.001*** | 0.298 p=.015* | 0.393 p=.001*** | 0.224 p=.073 | -0.044 p=.728 | 0.195 p=.111 |
| 3 | -- | -- | -- | -- | -- | -- | -- | -- | -- |
| 4 | 0.307 p=.051 | 0.358 p=.018* | 0.28 p=.076 | 0.404 p=.008 | 0.183 p=.246 | 0.184 p=.255 | 0.38 p=.016* | 0.211 p=.18 | 0.213 p=.181 |
| 5 | 0.111 p=.478 | 0.149 p=.33 | -0.097 p=.53 | 0.294 p=.05* | 0.137 p=.394 | 0.134 p=.381 | 0.203 p=.209 | 0.47 p=.002* | 0.201 p=.19 |
| 6 | 0.359 p=.001*** | 0.092 p=.412 | 0.238 p=.032* | 0.064 p=.57 | 0.35 p=.002* | 0.182 p=.109 | 0.277 p=.017* | 0.278 p=.013* | 0.491 p<.001*** |
| 7 | -- | -- | -- | -- | -- | -- | -- | -- | -- |
| 8 | 0.272 p=.067 | -0.099 p=.516 | -- | 0.142 p=.342 | 0.108 p=.477 | 0.215 p=.152 | 0.358 p=.018* | 0.232 p=.12 | 0.203 p=.175 |
| 9 | 0.471 p=0.023* | 0.443 p=0.03* | 0.349 p=0.103 | 0.335 p=0.118 | 0.274 p=0.195 | 0.291 p=0.167 | 0.337 p=0.125 | 0.105 p=0.635 | 0.088 p=.683 |
| 10 | 0.394 p=.069 | 0.485 p=.026* | 0.381 p=.072 | 0.109 p=.63 | 0.394 p=.063 | 0.099 p=.645 | 0.492 p=.02* | 0.231 p=.288 | 0.483 p=.017* |
| 11 | 0.462 p<.001*** | 0.41 p<.001*** | 0.415 p<.001*** | 0.449 p<.001*** | 0.41 p=.001*** | 0.402 p=.001*** | 0.286 p=.025* | 0.272 p=.026* | 0.316 p=.008* |
| 12 | 0.194 p=.296 | 0.433 p=.013* | 0.34 p=.057 | 0.34 p=.057 | 0.21 p=.25 | 0.189 p=.293 | 0.222 p=.23 | -0.233 p=.2 | 0.052 p=.779 |
| 13 | 0.375 p<.001*** | 0.409 p<.001*** | 0.269 p=.011* | 0.204 p=.056 | 0.456 p<.001*** | 0.224 p=.036* | 0.115 p=.296 | 0.208 p=.053 | 0.274 p=.011* |
| 14 | 0.243 p=.055 | 0.16 p=.211 | 0.143 p=.254 | 0.11 p=.384 | 0.344 p=.005* | 0.367 p=.003* | 0.041 p=.754 | 0.278 p=.025* | 0.211 p=.095 |
| 15 | 0.206 p=.346 | 0.13 p=.574 | 0 p=1 | 0.172 p=.432 | 0.01 p=.964 | -0.031 p=.888 | -0.084 p=.724 | -0.396 p=.068 | -0.314 p=.144 |
| 16 | -- | -- | -- | -- | -- | -- | -- | -- | -- |
| 17 | -- | -- | -- | -- | -- | -- | -- | -- | -- |
| 18 | -0.021 p=.93 | -- | 0.212 p=.369 | -- | 0.441 p=.051 | 0.176 p=.458 | -- | -0.016 p=.947 | 0.416 p=.068 |
| 19 | 0.299 p<.001*** | 0.426 p<.001*** | 0.21 p=.015* | 0.358 p<.001*** | 0.442 p<.001*** | 0.281 p=0.001*** | 0.398 p<.001*** | 0.404 p<.001*** | 0.393 p<.001*** |
| 20 | 0.337 p<.001*** | 0.256 p=.006* | 0.159 p=.088 | 0.183 p=.05* | 0.486 p<.001*** | 0.321 p<.001*** | 0.289 p=.002* | 0.301 p=.001*** | 0.38 p<.001*** |
| 21 | 0.283 p=.18 | 0.378 p=.068 | 0.376 p=.07 | 0.376 p=.07 | 0.579 p=.004* | 0.56 p=.005* | 0.114 p=.614 | 0.305 p=.167 | 0.393 p=.064 |

^a^ *Correlations with less than 20 cases have been removed.*

^b^ *ID: SWAN-OM item*

^c^ *ESQ items: 1: I feel that the person who saw me listened to me; 2: It was easy to talk to the person who saw me; 3: I was treated well by the person who saw me; 4: My views and worries were taken seriously; 5: I feel the people on Kooth know how to help me; 6: I have been given enough explanation about the help available on Kooth; 7: I feel that the people who have seen me on Kooth are working together to help me; 8: If a friend needed this sort of help, I would suggest to them to use Kooth; 9: Overall, the help I have received on Kooth is good.*

**Supplementary Table 6.** Pairwise correlations between individual SWAN-OM items and individual YCIS

|  |  | YCIS Items |  |  |
| --- | --- | --- | --- | --- |
| ID | SWAN-OM Item | 1 | 2 | 3 |
| 1 | Be comfortable asking for help outside Kooth | 0.394 p<.001*** | 0.513 p<.001*** | 0.444 p<.001*** |
| 2 | Find information about how to keep myself safe | 0.431 p<.001*** | 0.535 p<.001*** | 0.301 p=.013* |
| 3 | Feel safe in my relationships | -- | -- | -- |
| 4 | Be able to open up to people in my life | 0.639 p<.001*** | 0.561 p<.001*** | 0.554 p<.001*** |
| 5 | Talk about something I haven't told anyone before | 0.241 p=.111 | 0.326 p=.029* | 0.331 p=.026* |
| 6 | Feel listened to | 0.451 p<.001*** | 0.397 p<.001*** | 0.411 p<.001*** |
| 7 | Find out how useful it is to talk to someone | -- | -- | -- |
| 8 | Identify a solution to a problem in my life | 0.375 p=.009* | 0.608 p<.001*** | 0.463 p=.001*** |
| 9 | Learn how to feel better | 0.518 p=.01* | 0.603 p=.002* | 0.492 p=.015* |
| 10 | Learn the steps to achieve something I want | 0.798 p<.001*** | 0.798 p<.001*** | 0.635 p=.001*** |
| 11 | Explore how I feel | 0.686 p<.001*** | 0.586 p<.001*** | 0.552 p<.001*** |
| 12 | Be more comfortable with my feelings | 0.597 p<.001*** | 0.551 p=.001*** | 0.501 p=.003* |
| 13 | Understand my feelings and/or behaviours | 0.636 p<.001*** | 0.458  p<.001*** | 0.429 p<.001*** |
| 14 | Identify ways to help me worry less | 0.613 p<.001*** | 0.648  p<.001*** | 0.534 p<.001*** |
| 15 | Explore difficulties in my relationships | 0.249 p=.241 | 0.423 p=.04* | 0.614 p=.001*** |
| 16 | Learn how to relate to other people | -- | -- | -- |
| 17 | Learn how to manage conflict with others | -- | -- | -- |
| 18 | Identify solutions to improve my relationships | 0.153 p=.52 | 0.305 p=.191 | 0.729 p<.001*** |
| 19 | Feel better | 0.6 p<.001*** | 0.539 p<.001*** | 0.648 p<.001*** |
| 20 | Find ways I can help myself | 0.58 p<.001*** | 0.565 p<.001*** | 0.488 p<.001*** |
| 21 | Free text | 0.484 p=0.016* | 0.718 p<.001*** | 0.651 p=0.001*** |

^a^ Correlations with less than 20 cases have been removed.

^b^*YCIS items: 1, I now understand my feelings better. 2, I now have a better idea about how I can deal with my problems. 3, I now understand better what my strengths are.*

**Supplementary Table 7.** Pairwise Spearman-Rank correlation matrix between individual SWAN-OM items. ^a^

|  | Theme A | | Theme B | | | | | | Theme C | |
| --- | --- | --- | --- | --- | --- | --- | --- | --- | --- | --- |
| ID | 1 | 2 | 3 | 4 | 5 | 6 | 7 | 8 | 9 | 10 |
| 1 | 1 |  |  |  |  |  |  |  |  |  |
| 2 | 0.519 p=.013* | 1 |  |  |  |  |  |  |  |  |
| 3 | -- | -- | 1 |  |  |  |  |  |  |  |
| 4 | -- | -- | -- | 1 |  |  |  |  |  |  |
| 5 | -- | -- | -- | -- | 1 |  |  |  |  |  |
| 6 | -- | -- | -- | 0.236 p=.245 | 0.451 p=.008* | 1 |  |  |  |  |
| 7 | -- | -- | -- | -- | -- | -- | 1 |  |  |  |
| 8 | -- | -- | -- | -- | -- | 0.354 p=.055 | -- | 1 |  |  |
| 9 | -- | -- | -- | -- | -- | -- | -- | -- | 1 |  |
| 10 | -- | -- | -- | -- | -- | -- | -- | -- | -- | 1 |
| 11 | -- | -- | -- | -- | -- | -- | -- | -- | -- | -- |
| 12 | -- | -- | -- | -- | -- | -- | -- | -- | -- | -- |
| 13 | 0.464 p=.015* | -- | -- | -- | -- | 0.559 p=.008* | -- | -- | -- | -- |
| 14 | -- | -- | -- | -- | -- | -- | -- | -- | -- | -- |
| 15 | -- | -- | -- | -- | -- | -- | -- | -- | -- | -- |
| 16 | -- | -- | -- | -- | -- | -- | -- | -- | -- | -- |
| 17 | -- | -- | -- | -- | -- | -- | -- | -- | -- | -- |
| 18 | -- | -- | -- | -- | -- | -- | -- | -- | -- | -- |
| 19 | 0.536 p=.001*** | 0.559 p=.003* | -- | -- | -- | 0.723 p<.001*** | -- | -- | -- | -- |
| 20 | 0.392 p=.024* | 0.487 p=.006* | -- | -- | -- | -- | -- | -- | -- | -- |
| 21 | -- | -- | -- | -- | -- | -- | -- | -- | -- | -- |

Supplementary Table 7 (Continued)

|  | Theme D | | | | Theme E | | | | Theme F | | NA |
| --- | --- | --- | --- | --- | --- | --- | --- | --- | --- | --- | --- |
| ID | 11 | 12 | 13 | 14 | 15 | 16 | 17 | 18 | 19 | 20 | 21 |
| … | … | … | … | … | … | … | … | … | … | … | … |
| 11 | 1 |  |  |  |  |  |  |  |  |  |  |
| 12 | -- | 1 |  |  |  |  |  |  |  |  |  |
| 13 | 0.36 p=.027* | 0.619 p=.002* | 1 |  |  |  |  |  |  |  |  |
| 14 | 0.502 p=.006* | -- | 0.594 p<.001*** | 1 |  |  |  |  |  |  |  |
| 15 | -- | -- | -- | -- | 1 |  |  |  |  |  |  |
| 16 | -- | -- | -- | -- | -- | 1 |  |  |  |  |  |
| 17 | -- | -- | -- | -- | -- | -- | 1 |  |  |  |  |
| 18 | -- | -- | -- | -- | -- | -- | -- | 1 |  |  |  |
| 19 | 0.472 p=.015* | -- | 0.542 p=.002* | 0.557 p=.001*** | -- | -- | -- | -- | 1 |  |  |
| 20 | -- | -- | -- | 0.591 p=.003* | -- | -- | -- | -- | 0.648 p<.001*** | 1 |  |
| 21 | -- | -- | -- | -- | -- | -- | -- | -- | -- | -- | 1 |

^a^ *Most analyses could not be computed as pairwise cases were lower than 20.*
