# Supplementary material for: Examining concurrent validity and item selection of the Session Wants and Needs Outcome Measure (SWAN-OM) in a children and young people web-based therapy service
Source: Front Psychiatry. 2023 Feb 9;14:1067378. doi: 10.3389/fpsyt.2023.1067378 (PMC9947788; doi:10.3389/fpsyt.2023.1067378)
Supplement: Supplementary file 2 [file Data_Sheet_1.docx]

Supplementary Material

# Appendix

**Session Wants And Needs Outcome Measure [SWAN-OM] ^a b^.**

| **Theme**  *“Why did you come to chat today?”* | **ID** | **Pre-chat item selection**  *“Can you share more detail? Select up to 3 options”* | **Post-chat item scoring**  *“Did your chat support you in the way you hoped?”* ^b^ |
| --- | --- | --- | --- |
| A: ‘Understand what help I can get’ | | | |
|  | 1 | Be comfortable asking for help outside Kooth | I am now more comfortable asking for help outside Kooth |
|  | 2 | Find information about how to keep myself safe | I got some information about how to keep myself safe |
| B: ‘*Share my story with someone*’ | | | |
|  | 3 | Feel safe in my relationships | I now feel safer in my relationships |
|  | 4 | Be able to open up to people in my life | I now feel more able to open up to people in my life |
|  | 5 | Talk about something I haven't told anyone before | I talked about something personal to me |
|  | 6 | Feel listened to | I felt listened to |
|  | 7 | Find out how useful it is to talk to someone | It was useful to talk to someone |
|  | 8 | Identify a solution to a problem in my life | I have identified a possible solution to a problem in my life |
| C: ‘Set and achieve my goals’ | | | |
|  | 9 | Learn how to feel better | I have learnt some ways to feel better |
|  | 10 | Learn the steps to achieve something I want | I understand the steps to achieve my goal |
| D: ‘*Explore my emotions’* | | | |
|  | 11 | Explore how I feel | I was able to explore how I feel |
|  | 12 | Be more comfortable with my feelings | I feel more comfortable about my feelings now |
|  | 13 | Understand my feelings and/or behaviours | I understand my feelings and/or behaviours better |
|  | 14 | Identify ways to help me worry less | I have identified some ways to help me worry less |
| E: ‘Improve my relationships’ | | | |
|  | 15 | Explore difficulties in my relationships | I was able to explore some of the difficulties in my relationships |
|  | 16 | Learn how to relate to other people | I have learnt new ways to relate to other people |
|  | 17 | Learn how to manage conflict with others | I have learnt some ways to manage conflict with others |
|  | 18 | Identify solutions to improve my relationships | I have identified some solutions to improve my relationships |
| F: ‘Learn ways to cope’ | | | |
|  | 19 | Feel better | I was able to start feeling better |
|  | 20 | Find ways I can help myself | I have identified some ways to help myself |
| NA. Personalization item | | | |
|  | 21 | “Free-text” response | I choose my own focus before the chat |

^a^ *NA: Not applicable.*

^b^ *(1-5 Likert scale): [-2] strongly disagree, [-1] disagree [0] neither agree or disagree, [1] agree, [2] strongly agree*
